# Participatory Action Research: Meaningful Student Involvement in Medical Curriculum Development

**DOI:** 10.1111/tct.70431

**Published:** 2026-04-28

**Authors:** Yasmin Tyson, Muna Al‐Jawad

**Affiliations:** ^1^ Medical Student, Brighton and Sussex Medical School (BSMS) Brighton UK; ^2^ Senior Lecturer in Medical Education, Brighton and Sussex Medical School (BSMS) Brighton UK

**Keywords:** curriculum development, participation, partnership, undergraduate medicine

## Abstract

**Introduction:**

Our medical school aimed to involve students in changes to the Year 4 curriculum. We had feedback from graduates; however, there was little guidance available on how to meaningfully involve students. Most literature regarding curriculum co‐design in medical schools are case reports, with few using students in partnership such as in participatory action research (PAR). We used this process to determine how best to involve students in curriculum design.

**Methods:**

We recruited Year 4 medical students to discuss their experiences of the current curriculum, their opinions on potential new curriculum options and their reflections on the experience of this co‐design process. The PAR approach allowed researchers and participants to work in partnership to identify and analyse themes raised. We analysed transcripts of focus groups using reflexive thematic analysis.

**Results:**

Six students attended three focus groups, sharing congruent experiences from diverse perspectives. We considered students' perspectives on partnership working in curriculum design in terms of three major relationships: the student–faculty relationship, the relationship to curriculum and the peer‐to‐peer relationship. We discuss these findings in relation to pedagogic and feminist theories and use Arnstein's ladder of citizen participation to assess our current and prospective level of involvement.

**Conclusion:**

Previous research has shown that involving students in curriculum design has benefits for both the students and the teaching institution. Our action‐research project allowed the review of a potential new curriculum by the students and combined practice and theory to generate guidelines to be used for future involvement of medical students in curriculum co‐design.

## 1 Introduction

Multiple forces, from General Medical Council (GMC) requirements to pedagogical trends, shape the increasingly complex courses that train students to be doctors. As higher education is moving to view students as consumers [1], and there are fewer applications to an increasing number of places at medical school [2, 3], student feedback, such as in the National Student Survey (NSS), has never been more important.

Local NSS feedback from the 2024/2025 academic year was largely positive; 81.8% of students felt there were opportunities to give feedback on the course [4]. However, only 52.8% felt that students' feedback is clearly acted on [4], and 53.5% felt that changes to teaching on the course were not well communicated [4].

A recent scoping review of partnership working with students to co‐design health professions curricula suggested that despite a desire to include students as partners in the process of curriculum design, many were not able to navigate power relations for meaningful co‐design [5].

Curriculum is more than a plan of the course of study, and understanding this is key to exploring worthwhile collaboration. There are several aspects to curriculum design: espoused curriculum describes the values the University intends to impart, often expressed verbally or written in planning [6], in which outside forces such as the GMC and wider University have power; enacted curriculum is the physically expressed curriculum that is performed [6], in which teachers have the power to change; and the experienced curriculum, which is what the students truly live, and where the students are the experts. In the last, students view the curriculum through the lens of their own experience and, as such, each experiences the curriculum differently.

Alongside content, the curriculum outlines pedagogy. Positivist pedagogy relies heavily on empiricism, a behavioural objectives model [7], with the view that all useful attainments are measurable [7]. The *Flexner Report* [8] led to the creation of a behavioural objectives model, which has remained a perceived standard in medical education, especially in the United States [9]. As this model [7, 8] focuses on the quantifiable, objectives become distorted, and qualitative aspects, such as values and emotions, are lost [10]. Flexner allowed for no explicit consideration of race, gender and class in the ‘ideal doctor’ [11]. We used Freire's [12] work on education as a form of liberation to reject this positivist pedagogy. His work, outlined below, provided the moral basis of both our curriculum development [10] and partnership work.

Freire uses the banking model of education to describe positivist pedagogy [12]—a model where teachers deposit knowledge into passive students. In *Pedagogy of the Oppressed* [12], he argues against this model, instead advocating for a liberating pedagogy and conscientisation, this being a reciprocal relationship between teachers and students, which can only happen if the oppressed are conscious of their oppression. The liberation of the oppressed to understand their social context, Freire posits, allows for the humanisation of both the oppressor and the oppressed, and a critical dialogue can be established between them [12].

We believe that the conscientisation of students is only truly achieved when they are also involved in curriculum development. Participatory action research (PAR) and Freire's work both support student involvement in academic processes without the expected hierarchies. PAR empowers both researchers and participants to work in partnership and invoke change through a reflexive process of understanding both the situations they find themselves in and their relationship to them [13]. This collaboration encourages participants to not only participate in the research for change but also to be a driver for the change themselves [14].

Sherry R. Arnstein [15] presented a model ‘ladder’ to understand citizen involvement in decision making, ranging from nonparticipation to citizen power. She argues that a higher ‘rung’ of participation will lead to more meaningful engagement [15]. Arnstein's ladder still holds value when discussing partnership in education [16], and it is around this model that we will frame our results. First, we will discuss where we currently sit on the ladder with regards to student partnership, then we will consider where we can reasonably aim for on the ladder with our suggested changes.

Reflexivity is key to the practice of PAR; it necessitates us thinking about our own values and assumptions, and how these impact our topic of research [14]. For YT, this was largely based around experiences as a Year 4 student and, more generally, as a mature medical student from a widening participation background. For MAJ, this was an awareness of a position of power in curriculum change, but also the limits of this power, for example, if the team and students were not supportive of change.

The aim of this study is to describe and critically explore an alternative method of curriculum design whereby students are involved in partnership rather than viewed as consumers. We outline the process of student involvement rather than focussing on the specific aspects of the curriculum changing.

*The aim of this study is to describe and critically explore an alternative method of curriculum design whereby students are involved in partnership rather than viewed as consumers.*

## 2 Materials and Methods

We take a relativist ontological approach, understanding that reality is dependent on our interpretation and knowledge of it [17]. This is furthered by our constructionist epistemological assumptions, appreciating that knowledge is not an objective reflection of reality but of how we come to understand reality [17]. These assumptions allow us to most effectively use PAR, acknowledging that in this collaboration, we are involving different bases of knowledge from researchers and participants. We built on our relativist constructionist standpoint by approaching this project through an intersectional feminist lens. We wanted to go beyond superficial descriptions of the data to deeply understand the underlying power structures [18], with a view to future change.

The action and observation phases of this project happened concurrently. MAJ leads the course Curriculum Development Group (CDG), which consists of module leads, year leads and senior faculty who teach or lead the undergraduate course, alongside staff from professional services, library and technology enhanced learning teams. Members of CDG had started planning potential new curricula in response to the NSS and feedback from current Year 4 and Year 5 students, whilst YT and the other participants were experiencing the current Year 4 curriculum in real time. Evaluation from MAJ and the CDG, as well as YT and the participants, facilitated multiperspective reflexive practice.

Ethics approval for the project was gained from the medical school's Research Governance and Ethics Committee (Ref: ER/BSMS3192/7).

Year 4 medical students, recruited via convenience sampling [17], attended three focus groups. Focus groups were used rather than interviews as they allowed for exploration of shared perspectives [17]. YT facilitated focus groups without faculty present. The focus groups were audio‐recorded and transcribed. Participants were anonymised with pseudonyms, and identifying factors were removed or altered.

In keeping with our intersectional feminist standpoint, participants were asked to self‐identify in their own words any characteristics they were comfortable sharing, which included protected characteristics if they wished [19].

The first focus group was an open feedback forum of participants' views on the current Year 4 curriculum, which were then summarised. Based on these views and ongoing work from the CDG, we provided three options for a potential new Year 4 curriculum. We discussed each of these options in the second focus group. In the final focus group, we discussed participants' experiences of being involved in curriculum development. This served as member checking [20], where we asked participants to check the accuracy and resonance of our ideas for curriculum development and themes.

The third focus group transcript was coded by YT using reflexive thematic analysis [17]. We used inductive coding [21], in keeping with our goals in PAR. However, we recognise that codes will be influenced by the theoretical and social positions of the researchers [21]. To ensure quality, codes were triangulated with MAJ's separate analysis of the transcript, following the same approach.

During the project, YT also attended CDG meetings twice (as an observer and participant) and observed other meetings where students are involved in curriculum development. These were a phase forum (where students meet with phase leads to discuss issues with their experience of the course) and a meeting of the Inclusive Practice Partners (a group of students paid to work in partnership with faculty on decolonising the curriculum). These observations were used to more fully understand the different ways that students are involved in curriculum development on the course, and to help interpret focus group data in this light. Both YT and MAJ kept research journals throughout the project to express and guide our criticality and reflexivity.

## 3 Results

Six Year 4 students took part in the focus groups. Participant demographics, including YT as the moderator, are included verbatim below, alongside their pseudonym (a colour).

We recognise that the identities of our participants do not make them representatives of these groups as a whole.

- **Orange:** white cisgender female, queer, graduate student
- **Purple:** mixed race, queer, cisgender female, has a disability, came straight from school
- **Blue:** graduate, queer cis white woman, has a long‐term condition, disabled
- **Pink:** mixed race, cisgender female, straight, international student, came straight out of school, not disabled
- **Yellow:** straight white cisgender, graduate, has dyslexia
- **Green:** East African cisgender queer male, came straight from school, able‐bodied
- **Moderator:** white cisgender female, has a long‐term illness, widening participation student, mature student

We developed our themes by combining inductive coding with supporting literature from feminism and liberating pedagogy, both of which hold relationships centrally. Three themes are described, with a brief introduction to the related theory. The themes relate to the different relationships experienced by students within curriculum development. Each theme has been split into subthemes and is supported by participant quotes.

### 3.1 Theme 1: Student–Faculty Relationship

Feminist thinkers [18, 22, 23] are concerned with the relationships within power structures. We recognise the University as reinforcing a traditional student–faculty hierarchy [10], which was noted as important to students as we analysed the data. Within the student–faculty relationship in the context of curriculum design, we noted recurring subthemes of communication, roles and role clarity, respect and disrespect and power and hierarchy.

#### 3.1.1 Communication

Yellow and Orange discussed how the focus groups allowed for indirect yet effective communication between participants and CDG faculty. Yellow emphasised the importance of demonstrating that they had been listened to:

But I think Orange's right in that it was nice that we said the things that we found difficult in the first session and then the second session it was like it had actually been listened to and these were the options. **(Quote 1; Yellow)**

Orange suggested that if a change were to be implemented to Year 4, those in years imminently affected by the change should be involved as well. She based this on her previous experiences of poor communication regarding curriculum structure:

But I also think it'd be good to talk to the year twos and year threes about the changes because I think every year that I've gone into on this degree I've felt like I've gone in blind and I haven't really understood like the structure or what to do where to go so it'd be really helpful. **(Quote 2; Orange)**

#### 3.1.2 Roles and Role Clarity

Orange made it clear that she was aware of not being an expert in curriculum design, and that receiving justified negative responses would not be a problem:

… Because if all the ideas we've come up with have come from quite a naïve space where we don't really like get the logistics I don't mind being told that actually “Your ideas are a bit weak” but I like to feel like I've been heard. **(Quote 3; Orange)**

Yellow illustrated her understanding of their roles as participants in this project, acknowledging students' limited understanding of the practicalities of curriculum design. She mentioned this in relation to the clarity provided by YT and MAJ from the outset of the project:

And also … you were quite clear that even in the future what we say it might not be able to be that they can implement it because like we're saying from one level but they're saying from the level of actually developing it and what's possible so yeah I think from the get‐go it's been very clear. **(Quote 4; Yellow)**

Despite this perceived clarity, there were still instances where students acted outside of their defined roles. One participant said that an idea put to them would not work, as the curriculum team would not be able to deliver it. This was outside of their remit, as they had little understanding of the feasibility of delivery of a model of teaching.

#### 3.1.3 Respect and Disrespect

On being asked their opinions on having a member of faculty present in the focus groups, Orange commented on how this may limit participants' ability to speak freely for fear of transgressing the unspoken respect from medical students to doctors:

I think it would really change the tone of things to have somebody in here cos I think all of us would be very uncomfortable with critiquing or even if it was somebody and we knew that they were a paediatrician we might not be comfortable being like “we found the paediatric doctors really patronising.” **(Quote 5; Orange)**

This references an existing hierarchy and power dynamic between students, addressed by both clinical and other medical school staff in the *power and hierarchy* subtheme.

#### 3.1.4 Power and Hierarchy

Purple stated explicitly that she would not have participated in the project if a member of faculty had been present in the focus groups:

If there was somebody that was going to be here I wouldn't have come to these sessions like it would've just freaked me out completely. **(Quote 6; Purple)**

Green acknowledged the subtleties of this pre‐existing power dynamic, including having to be conscious of communications:

There's a lot of like ways you have to structure your answer when you talk to a superior. **(Quote 7; Green)**

Green also discussed the importance of anonymity in relation to this conscious communication. He reflected that this allowed for honest speech without having to intentionally ‘improve’ his opinions for anyone.

### 3.2 Theme 2: Relationship to Curriculum and Curriculum Change

Our second theme was shaped by considering students' reactions to a curriculum heavily influenced by positivism [8]. Our data clearly showed that they valued the chance to influence the curriculum in line with liberating pedagogy [12]. Participants' experiences with their relationship to curriculum and curriculum change highlighted the importance of an adult relationship, treating with respect and experts by experience.

#### 3.2.1 Adult Relationship

Whilst reflecting on the progression of the focus groups, specifically the second group following feedback from the CDG, Yellow shared how this channel of communication was appreciated as an adult‐to‐adult relationship:

That also makes it feel like they're talking to us adult to adult … because if it's just “no we can't do that” well then why … like it makes it seem like we're being talked down to as children like they don't … believe that we deserve to know why … just getting an explanation as to why just feels a lot more … better as a conversation. **(Quote 8; Yellow)**

Later in the same discussion, Yellow and Blue further explored how being treated as adults allowed them to align themselves with the CDG's views, understanding their espoused values:

Yeah and hearing when you say that there are some resistances to this hearing why the resistances are there so the fact that there's fears that … people will slip through the cracks and be more likely to fail it's like okay that actually makes sense yeah and it's okay … the resistances are because they're trying to kind of like. Blue: ((in overlap)) ‘Support people’. **(Quote 9; Yellow and Blue)**

These points both expand on the previously highlighted importance of effective communication between faculty and students and demonstrate the positive impact effective communication can have.

#### 3.2.2 Treating With Respect

In keeping with the subject of being part of an adult relationship, Orange shared her thoughts on the right of medical students to be treated with respect by faculty:

But just like … to be treated with respect and be treated like we actually have a say because it's not an item that we're given it's essentially an experience and if we have feedback about how the experience should be run that should be heard. **(Quote 10; Orange)**

#### 3.2.3 Experts by Experience

On the topic of which students to involve in curriculum development, YT shared thoughts from a conversation with MAJ about getting feedback from different demographic groups:

… The more people you have with … things like learning differences or LSPs [learning support plans] or different ways of coming into medicine quite often the more slightly more marginalised give more in depth feedback and if they're the ones who're … more likely to struggle with the course then getting their feedback and addressing it is more likely to benefit everyone. **(Quote 11; Moderator)**

Yellow identified again the importance of communication between faculty and students, and how doing so poorly further drives differences between espoused and experienced curriculum:

Like I think they come up with such great ideas of how they can help us but they don't actually talk to us … so we can't then say “yes that is helpful” or “that would be helpful at this point.” **(Quote 12; Yellow)**

### 3.3 Theme 3: Peer‐to‐Peer Relationship

We constructed our third theme considering the power of solidarity amongst students. This was highlighted from a feminist perspective in the writings of Ahmed [24], and from a curriculum standpoint by Henry Giroux [25, 26]. The relationship between participants, especially when discussing their shared experiences, was empowering. We noticed key subthemes here of being ignored by faculty and a sense of solidarity amongst medical students.

#### 3.3.1 Ignored by Faculty

Yellow reflected on one of the current methods of collecting student feedback—the phase forum. She highlighted that students do not often utilise them as intended by faculty due to shared experiences of feeling disregarded:

Cos I feel like … that's what the phase leader forums are for … but not everybody brings up all of their complaints at the phase leader forum cos they've often found that it's been shut down. **(Quote 13; Yellow)**

#### 3.3.2 Solidarity

Orange shared how the focus groups encouraged sharing negative experiences without feeling isolated by them, a feeling that was echoed by many throughout the third meeting:

… It can be quite hard to complain in isolation cos it's very easy to be like oh this is just a me problem … whereas having a group it's quite helpful to be like oh it might be a small thing and everyone else is like “no I'm thinking the same thing.” **(Quote 14; Orange)**

Yellow, Blue and Orange later brought up the importance of solidarity amongst participants again. Yellow compared the positive echo created in the focus groups to a more solitary method of feedback:

Yellow: ‘I wanna emphasise the solidarity … aspect of this compared to like filling out a feedback form is that you're less thinking “oh this just a me problem”’

Blue: ((in overlap)) ‘Yeah’

Orange: ((in overlap)) ‘Yeah’

Yellow: ‘That you're having everybody else be like “yeah I've struggled with that too” and that helped’. **(Quote 15; Yellow, Blue and Orange)**

Orange spoke about solidarity amongst medical students more broadly, not just as part of this project. She emphasised how this instinctive support can be harnessed to improve communication between students and faculty:

And I think I do think as a cohort … we don't want to see our other students fail and I think there is a solidarity within medical students we're not alike to other degrees in that we're like essentially kind of looking at our coworkers and we want to support people …. **(Quote 16; Orange)**

Orange also noted how this would help maintain the important adult relationship from faculty that is highly valued by students.

Blue and Purple discussed the positive impact that hearing similar experiences from other students had on them:

Blue: ‘I also think it's nice … to hear that other … people in other placements or other groups are also experiencing the same thing cos otherwise it feels like’

Purple: ‘It feels like we're being the difficult ones’. **(Quote 17; Blue and Purple)**

## 4 Discussion

**FIGURE 1:**
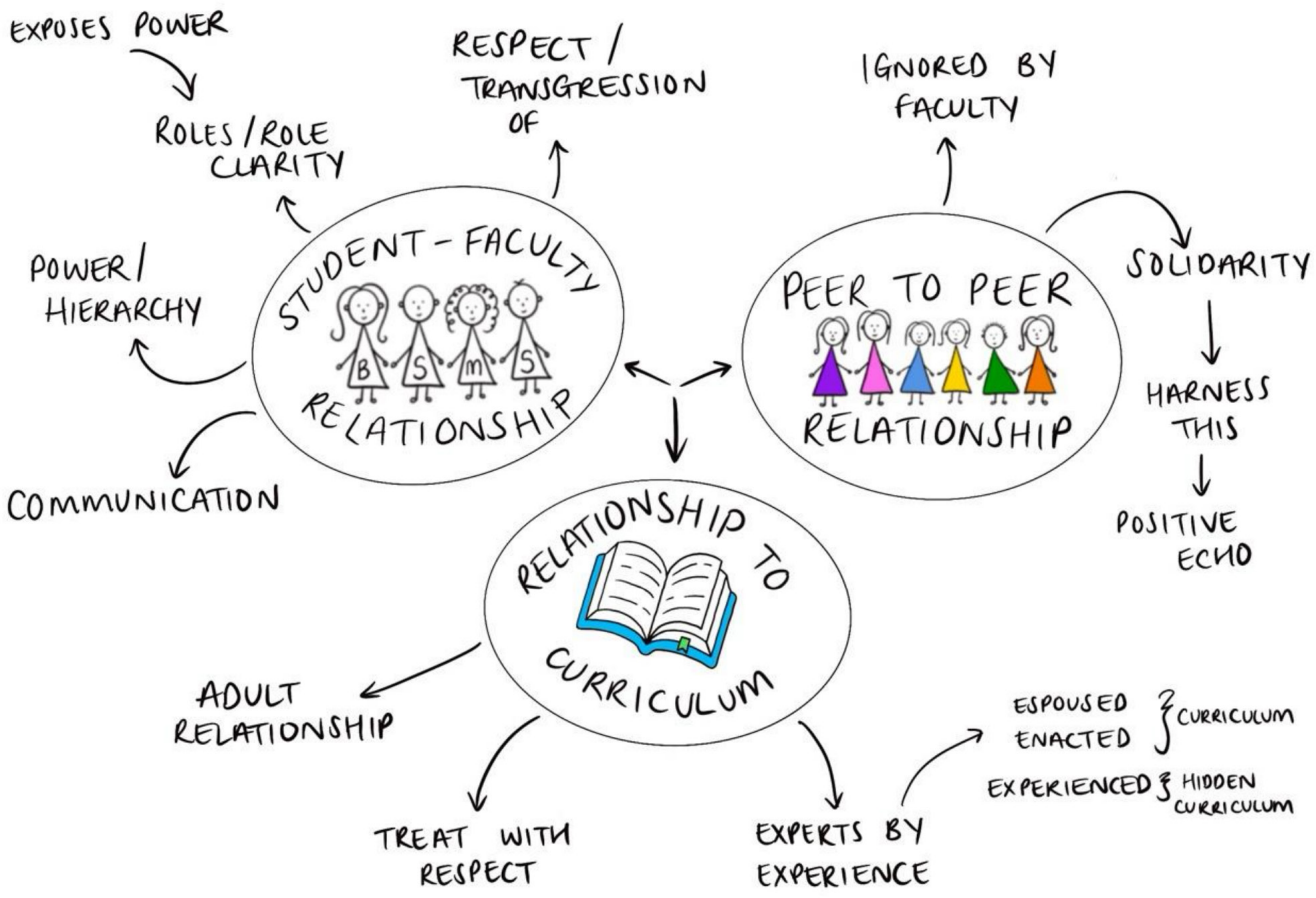
Key relationships and subthemes, drawn by YT.

As set out in Section 3, we identified three key relationships in the analysis of participants' experiences of being involved in curriculum development, as well as subthemes as part of these relationships (Figure 1).

### 4.1 Student–Faculty Relationship

In her book, *The Feminist Killjoy Handbook* [18], Ahmed describes the ‘feminist killjoy’ as one willing to challenge the status quo, even at the cost of being perceived as disruptive. Throughout our project, we all became feminist killjoys (or perhaps *more* feminist killjoys). With the support of MAJ and the CDG, we were able to foster a safe and comfortable environment in the focus groups without having to hear direct (possibly defensive) responses from faculty, so there were no direct responses to our disruption.

Participants unanimously agreed that a member of faculty should not have been present in focus group discussions (Quote 6), which, due to power dynamics, would have hindered our ability to be honest and tackle difficult conversations. This was inadvertently calling attention to the fact that norms such as having a member of faculty facilitate student feedback may actually be further marginalising voices [18]. An open dialogue allowed for critique of well‐established systems without fear of repercussion, exposing the power dynamics at play [18].

Bell hooks [22] builds on the critical pedagogy work of Paolo Freire [12] (detailed in *Relationship to curriculum & curriculum change*) by positioning education as a practice of freedom [22]. We followed Hooks' value of students being empowered to challenge oppressive structures and transgress boundaries [22]. We found that, although students often speak mercilessly about our experiences with each other, our most honest thoughts are not often given a platform to be addressed by those in power (Quotes 7 and 8).

Audre Lorde [23] famously conceded that we cannot solve oppression by working with oppression; ‘for the master's tool will never dismantle the master's house’ [23]. Despite working for ‘the master's house’, MAJ, in developing our project, was able to facilitate a safe space to foster critical thinking. Hooks acknowledges this as a crucial role of the teacher [22], as well as to utilise education to address emotions alongside intellect [22].

The reshaping of our relationship with faculty through this project felt empowering and an act of resistance. However, we remain conscious that this empowerment is influenced and favoured by aspects of our identities. As hooks says, ‘race, sex, and class privilege empower some students more than others, granting “authority” to some voices more than others’ [22]. An appreciation of intersectionality and solidarity can contribute to a change in medical culture [22]. Therefore, when students, especially marginalised ones, do transgress the ingrained hierarchy, the medical school should listen. As iterated by Yellow and Orange, to establish and maintain a good relationship, we need mutual respect and effective communication (Quotes 1 and 2).

*The reshaping of our relationship with faculty through this project felt empowering and an act of resistance. However, we remain conscious that this empowerment is influenced and favoured by aspects of our identities.*

### 4.2 Relationship to Curriculum and Curriculum Change

Participants' experiences with their relationship to curriculum and curriculum change highlighted the importance of an adult relationship, being treated with respect and the concept of experts by experience. Here, we will discuss both positivist [7, 8, 10] and critical pedagogies [12, 22], how they relate to our curriculum and our understanding of education.

Multiple participants identified as queer, a demographic known to be othering for medical students [27, 28]. Queer clinicians face discrimination and hostility in the workplace [29]. However, our participants' expertise by experience as not only medical students but also queer medical students carries weight and should be rightly valued and used by the medical school.

Positivist pedagogy [7, 8, 10] and Flexnerism [8, 9] place the teacher at the centre and judge standards of education on the espoused and enacted curriculum, excluding the experienced curriculum [10]. The experienced curriculum was significantly important to students; it allowed for the strengthening of an already present solidarity. Without acknowledging the experiences of the recently graduated students [4] and the participants, this project would not have happened. Faculty would have remained satisfied with their espoused values, unaware that students were not receiving what the CDG intended for their learning experience (Quote 12).

*Positivist pedagogy and Flexnerism places the teacher at the centre and judges standards of education on the espoused and enacted curriculum, excluding the experienced curriculum. The experienced curriculum was significantly important to students; it allowed for strengthening of an already present solidarity.*

In our project, as in PAR praxis generally, we have followed Freire's ideas [12]—by inviting students, including YT, into the usually hidden realm of curriculum design and encouraging us to address issues that affect our lives. The conscientisation occurred when we gained insight into the CDG workings and learned about the espoused curriculum (Quote 9). The humanisation [12] was evident from our side as students when we received explanations for why things in the enacted curriculum where the way they were (Quote 8). There was an almost instantaneous destruction of the dichotomy between the espoused and experienced curricula at this point; once we understood the values the CDG was prioritising (e.g., certain measures to be able to catch and help people potentially failing), our experiences of those actions were less negative.

In short, once we were introduced to a reciprocal adult relationship and treated with respect, we could adopt a more familiar and understanding relationship to the curriculum and those responsible for it.

This movement from viewing education as a positivist to a critical pedagogy allowed YT, as a student and participant, a moment of epistemological and personal reflexivity. YT's previous knowledge of education was not only expanded but also fundamentally changed to know that learning is socially constructed [12, 30], and, despite being a mature student, she did not need to be seen as ‘immature’ in the relationship solely because she is the student. As participants, we grew together to the understanding that how we come to know our knowledge is just as important as the knowledge itself.

### 4.3 Peer‐to‐Peer Relationship

The relationship between participants, especially when discussing their shared experiences, was empowering. We noticed key themes here of being ignored by faculty and a sense of solidarity amongst medical students which was demonstrated as a positive echo in the focus groups. Harnessing this solidarity provides an important opportunity when working with medical students.

Border pedagogy recognises various boundaries that distinguish an *us* and a *them* [25]; in this case, the *us* is the authority figure of the CDG and faculty, and we medical students are the isolated *them*. Giroux [26] acknowledges that educational contexts link to and reflect wider society, an ideology not possible in positivist pedagogy, as it does not recognise the link between knowledge and social control [10]. He also states that the students must cross borders as an act of transgression [26].

Orange reflected on how the sense of solidarity went further than just between the participants of the focus groups; that there is an ingrained sense of solidarity amongst all medical students, as they are future colleagues as well as classmates (Quote 16). By crossing the borders of our education now, we are solidifying that sense of solidarity. Harnessing this solidarity by empowering us to be involved in curriculum development now has the potential to make us stronger graduates in the future [31].

*By crossing the borders of our education now, we are solidifying that sense of solidarity. Harnessing this solidarity by empowering us to be involved in curriculum development now has the potential to make us stronger graduates in the future.*

Border pedagogy also recognises that individual differences do not negate a common struggle [25], and that as a collective, we do not have to assume our struggles are the same to feel solidarity [26]. The fact that we may not all agree on the best new Year 4 curriculum does not deny the fact that we are useful contributors to its inception. To us, this shows how intersectionality is not an inconvenience but a driving force and must be acknowledged and respected.

Sara Ahmed recognises that solidarity involves commitment and work [24]. Whenever the concept of creating a ‘positive echo’ of shared experiences came up in the focus groups, it was often coupled with a negative experience in the same context. For example, Yellow emphasised how much easier it was to share feedback in the focus groups compared to a feedback form (Quote 15). This shows that the commitment is already there; she was willing to give feedback via the form despite it leaving her feeling isolated. The phase leader forums were the most frequently mentioned as examples of students feeling ignored by faculty; however, students still attended. Students are already putting in the work driven by their solidarity; it is just not being actioned. This pre‐existing motive should be directed into effective movement.

Borders may shift to one side's benefit, usually the authoritative *us* [26]. Our project demonstrates how we can redefine borders to more equally benefit *them*. With a direct line to our experiences of curriculum, the faculty can aid in the progressive escalation of positive echo to solidarity to action. Although the pre‐existing solidarity amongst medical students is invaluable, it is not enough to rely on for change and must be built upon. As Ahmed says, ‘Solidarity has to be achieved, not assumed’ [18].

*Borders may shift to one side's benefit, usually the authoritative* us. *Our project demonstrates how we can redefine borders to more equally benefit* them.

### 4.4 Back on the Ladder

**FIGURE 2:**
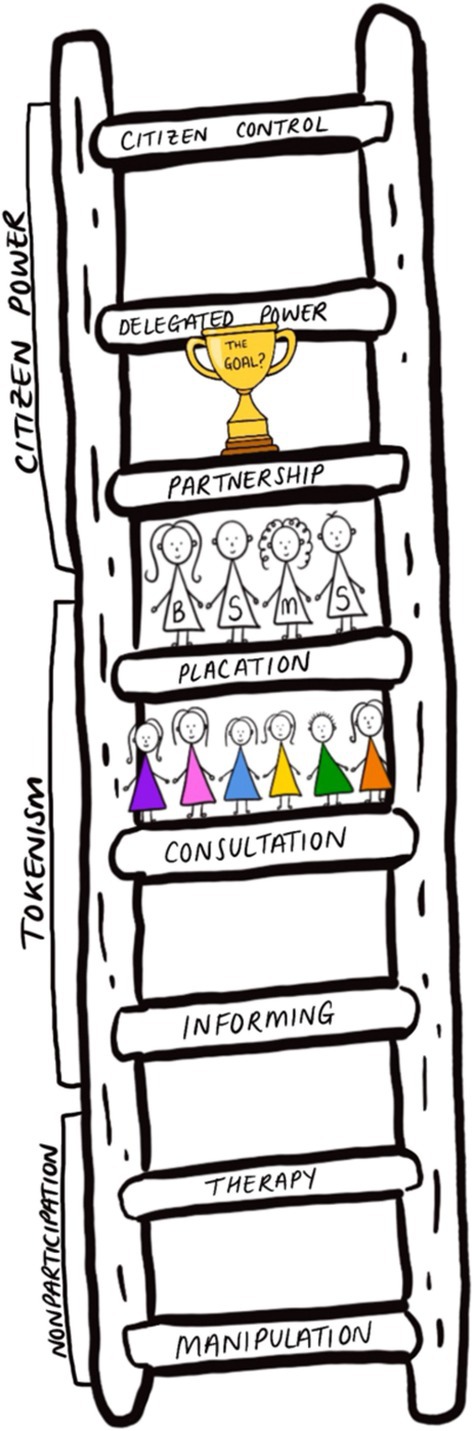
Arnstein's ladder, drawn by YT.

We believe that our course currently sits firmly in the middle, or tokenistic rungs of Arnstein's ladder [15]. Most interestingly, we have inferred that at present, there is a disparity between the CDG and the student body as to which exact rung we sit on (Figure 2).

From the student experiences, we see ourselves on the Consultation rung [15], where we are asked for feedback but not always taken seriously. Faculty, however, viewing the curriculum with their espoused values at the forefront, are more likely to see us on the Placation rung [15]; they retain the power to make the decisions but have given us a voice. Following the student participants' conscientisation, we can now see this gap. The first step to meaningful involvement will be to close this and achieve true Placation [15]. Only then could we consider Partnership [15] and whether it is achievable or even desirable in this context.

### 4.5 Limitations

Although PAR aims to involve participants in the analysis of data, due to time constraints and limited availability, we were unable to achieve this. Convenience sampling meant that we could not control the characteristics of our participants. In spite of this, our student group was relatively diverse.

We recognise that this study was conducted in a single institution with a small number of students, limiting its generalisability and transferability. However, we believe the themes identified may be useful for other institutions to consider.

An intersectional feminist lens was purposefully chosen for our analysis; however, we are aware that this blinds us to certain viewpoints, which may be relevant to some researchers.

The fact that YT is a medical student and MAJ is a member of faculty means that the analysis of the data will always be biased. We've mitigated this as much as possible through reflexivity and openness about our positions.

## 5 Conclusion

In this project, we aimed to critically explore the process of student involvement in curriculum development. We used data from a small project, redesigning Year 4 as a way to critique current views of curriculum from an intersectional feminist perspective. We provide guidance, based on our findings, on how to meaningfully involve medical students in curriculum development.

Our recommendations are as follows: Curriculum developers should understand the theory behind the curriculum. We suggest interrogating the espoused, enacted and experienced curricula and resisting positivist pedagogy to value student experiences.

Faculty should communicate with respect to facilitate an adult relationship and make time to build trust and true partnerships with students. Be clear from the outset what people's roles are, what they can and cannot expect to change and in what timescales, whilst still allowing students to move up the ladder of involvement.

Faculty and students should be aware of the power dynamic, and that collaboration requires students to become disruptors. Make adjustments to mitigate this power imbalance, such as student‐led focus groups. Allow students more power by educating them about curriculum development. Consider that purposeful engagement with marginalised groups may be more useful than a large random sample.

Finally, harness the students' solidarity; most medical students want to help each other and faculty, which facilitates transformative change.

## Author Contributions

**Yasmin Tyson:** conceptualisation (supporting); data curation; formal analysis (equal); investigation; methodology (supporting); project administration; resources (equal); visualisation; writing – original draft preparation; writing – review and editing (equal). **Muna Al‐Jawad:** conceptualisation (lead); formal analysis (equal); methodology (lead); resources (equal); supervision; validation; writing – review and editing (equal).

## Funding

The authors have nothing to report.

## Conflicts of Interest

The authors declare no conflicts of interest.

## Data Availability

The data that support the findings of this study are available from the corresponding author upon reasonable request.

## References

[1] M. Molesworth , E. Nixon , and R. Scullion , “Having, Being and Higher Education: The Marketisation of the University and the Transformation of the Student Into Consumer,” Teaching in Higher Education 14, no. 3 (2009): 277–287, 10.1080/13562510902898841.

[2] UCAS , “Cycle Applicant Figures—16 October Deadline,” (2024), https://app.powerbi.com/view?r=eyJrIjoiMzViMGYwNjktNGU2Ny00YzY3LTkxN2UtODQ1ZWU4YmM5NmQ1IiwidCI6ImM2MmJjYTQ0LTcwY2ItNDU3Yi1hMzU1LWRlYWE1Y2I3ZTY4OSIsImMiOjh9. [Cited 29th May 2025].

[3] NHS England , “NHS England Update on Medical School Training Places, 28 February Letter to Royal College of Physicians,” https://www.england.nhs.uk/long‐read/nhs‐england‐update‐on‐medical‐school‐training‐places‐28‐february‐letter‐to‐royal‐college‐of‐physicians/ [cited 29th May 2025].

[4] Office for Students , “National Student Survey Data: Provider‐Level Dashboard,” https://www.officeforstudents.org.uk/. [cited 16th March 2025].

[5] J. Abbonizio , C. Palermo , G. Brand , N. Buus , E. Fossey , and J. Dart , “Co‐Designing Formal Health Professions Curriculum in Partnership With Students: A Scoping Review,” Medical Teacher 47, no. 3 (2024): 413–424, 10.1080/0142159X.2024.2339403. 38621357

[6] M. M. Kennedy , “The Role of Preservice Teacher Education,” in Teaching as the Learning Profession: Handbook of Teaching and Policy, eds. L. Darling‐Hammond and G. Sykes (Jossey Bass, 1999), 54–86.

[7] D. Scott , “Behavioural Objectives and W.J. Popham,” in Critical Essays on Major Curriculum Theorists (Routledge, 2008), 21–30.

[8] C. B. Chapman , “‘The Flexner Report’ by Abraham Flexner,” Daedalus 103, no. 1 (1974): 105–117, https://www.jstor.org/stable/20024193. 11615417

[9] A. H. Beck , “The Flexner Report and the Standardization of American Medical Education,” Journal of the American Medical Association 291, no. 17 (2004): 2139–2140, 10.1001/jama.291.17.2139. 15126445

[10] H. Giroux , “Schooling and the Culture of Positivism: Notes on the Death of History,” Educational Theory 29, no. 4 (1979): 263–284, 10.1111/j.1741-5446.1979.tb00859.x.

[11] M. Bailey , “The Flexner Report: Standardizing Medical Students Through Region‐, Gender‐, and Race‐Based Hierarchies,” American Journal of Law & Medicine 43, no. 2–3 (2017): 209–223, 10.1177/0098858817723660. 29254466

[12] P. Freire , Pedagogy of the Oppressed (Penguin, 1996).

[13] F. Baum , C. MacDougall , and D. Smith , “Participatory Action Research,” Journal of Epidemiology and Community Health 60, no. 10 (2006): 854–857, 10.1136/jech.2004.028662. 16973531 PMC2566051

[14] S. Kjellström and A. Mitchell , “Health and Healthcare as the Context for Participatory Action Research,” Sage Journals 17, no. 4 (2019): 419–428, 10.1177/1476750319891468.

[15] S. R. Arnstein , “A Ladder of Citizen Participation,” Journal of the American Institute of Planners 35, no. 4 (1969): 216–224, 10.1080/01944366908977225.

[16] S. Varwell , “A Literature Review of Arnstein's Ladder of Citizen Participation: Lessons for Contemporary Student Engagement,” Exchanges: The Interdisciplinary Research Journal 10, no. 1 (2022): 108–144, 10.31273/eirj.v10i1.1156.

[17] V. Braun and V. Clarke , Successful Qualitative Research: A Practical Guide for Beginners (SAGE, 2013).

[18] S. Ahmed , The Feminist Killjoy Handbook (Allen Lane, 2023).

[19] UK Primary Legislation , “Equality Act 2010,” https://www.legislation.gov.uk/ukpga/2010/15/contents. [cited 29th May 2025].

[20] L. Birt , S. Scott , D. Cavers , C. Campbell , and F. Walter , “Member Checking: A Tool to Enhance Trustworthiness or Merely a Nod to Validation,” Qualitative Health Research 26, no. 13 (2016): 1802–1811, 10.1177/1049732316654870. 27340178

[21] V. Braun and V. Clarke , Thematic Analysis: A Practical Guide (SAGE, 2022), 55–56.

[22] B. Hooks , “Teaching to Transgress: Education as the Practice of Freedom,” Oxon: Routledge ; (1994).

[23] A. Lorde , “The Master's Tools Will Never Dismantle the Master's House,” in Sister Outsider: Essays and Speeches (Crossing Press, 1984).

[24] S. Ahmed , The Cultural Politics of Emotion (Edinburgh University Press, 2004).

[25] H. Giroux , “Border Pedagogy and the Politics of Postmodernism,” Social Text 28 (1991): 51–67, 10.2307/466376.

[26] H. Giroux , Pedagogy and the Politics of Hope (Routledge, 1997).

[27] H. Bintley , “Expression, Oppression and Queer Bodies: A Pilot Study Exploring the Lived Experiences of LGBTQ+ Medical Students in the UK,” Advanced Journal of Professional Practice 4, no. 1 (2023): 1–24, 10.22024/UniKent/03/ajpp.1125.

[28] D. Ly and R. Chakrabarti , “‘I'm Looking as White and as Straight as Possible at All Times’: A Qualitative Study Exploring the Intersectional Experiences of BAME LGBTQ+ Medical Students in the UK,” BMJ Open 14, no. 8 (2024): e086346, 10.1136/bmjopen-2024-086346. PMC11337697 39160106

[29] M. J. Eliason , S. L. Dibble , and P. A. Roberston , “Lesbian, Gay, Bisexual, and Transgender (LGBT) Physicians' Experiences in the Workplace,” Journal of Homosexuality 58, no. 10 (2011): 1355–1371, 10.1080/00918369.2011.614902. 22029561

[30] J. Dewey , The Child and the Curriculum (University of Chicago Press, 1902).

[31] A. Cook‐Sather and K. Matthews , “Practising Student Voice in University Teaching and Learning: Three Anchoring Principles,” Journal of University Teaching and Learning Practice 20, no. 6 (2023): 1–11, 10.53761/1.20.6.2.

